# The ankle dorsiflexion kinetics demand to increase swing phase foot-ground clearance: implications for assistive device design and energy demands

**DOI:** 10.1186/s12984-024-01394-x

**Published:** 2024-06-21

**Authors:** Soheil Bajelan, W. A. (Tony) Sparrow, Rezaul Begg

**Affiliations:** Institute for Health and Sport, Victoria University, Melbourne, Australia

**Keywords:** Ankle dorsiflexion moment, Ankle dorsiflexion energy, Ankle dorsiflexion muscle forces, Minimum foot clearance, Swing biomechanics, Ankle foot orthoses, Ankle assistive devices

## Abstract

**Background:**

The ankle is usually highly effective in modulating the swing foot’s trajectory to ensure safe ground clearance but there are few reports of ankle kinetics and mechanical energy exchange during the gait cycle swing phase. Previous work has investigated ankle swing mechanics during normal walking but with developments in devices providing dorsiflexion assistance, it is now essential to understand the minimal kinetic requirements for increasing ankle dorsiflexion, particularly for devices employing energy harvesting or utilizing lighter and lower power energy sources or actuators.

**Methods:**

Using a real-time treadmill-walking biofeedback technique, swing phase ankle dorsiflexion was experimentally controlled to increase foot-ground clearance by 4 cm achieved via increased ankle dorsiflexion. Swing phase ankle moments and dorsiflexor muscle forces were estimated using AnyBody modeling system. It was hypothesized that increasing foot-ground clearance by 4 cm, employing only the ankle joint, would require significantly higher dorsiflexion moments and muscle forces than a normal walking control condition.

**Results:**

Results did not confirm significantly increased ankle moments with augmented dorsiflexion, with 0.02 N.m/kg at toe-off reducing to zero by the end of swing. Tibialis Anterior muscle force incremented significantly from 2 to 4 N/kg after toe-off, due to coactivation with the Soleus. To ensure an additional 4 cm mid swing foot-ground clearance, an estimated additional 0.003 Joules/kg is required to be released immediately after toe-off.

**Conclusion:**

This study highlights the interplay between ankle moments, muscle forces, and energy demands during swing phase ankle dorsiflexion, offering insights for the design of ankle assistive technologies. External devices do not need to deliver significantly greater ankle moments to increase ankle dorsiflexion but, they should offer higher mechanical power to provide rapid bursts of energy to facilitate quick dorsiflexion transitions before reaching Minimum Foot Clearance event. Additionally, for ankle-related bio-inspired devices incorporating artificial muscles or humanoid robots that aim to replicate natural ankle biomechanics, the inclusion of supplementary Tibialis Anterior forces is crucial due to Tibialis Anterior and Soleus co-activation. These design strategies ensures that ankle assistive technologies are both effective and aligned with the biomechanical realities of human movement.

## Background

**Fig. 1:**
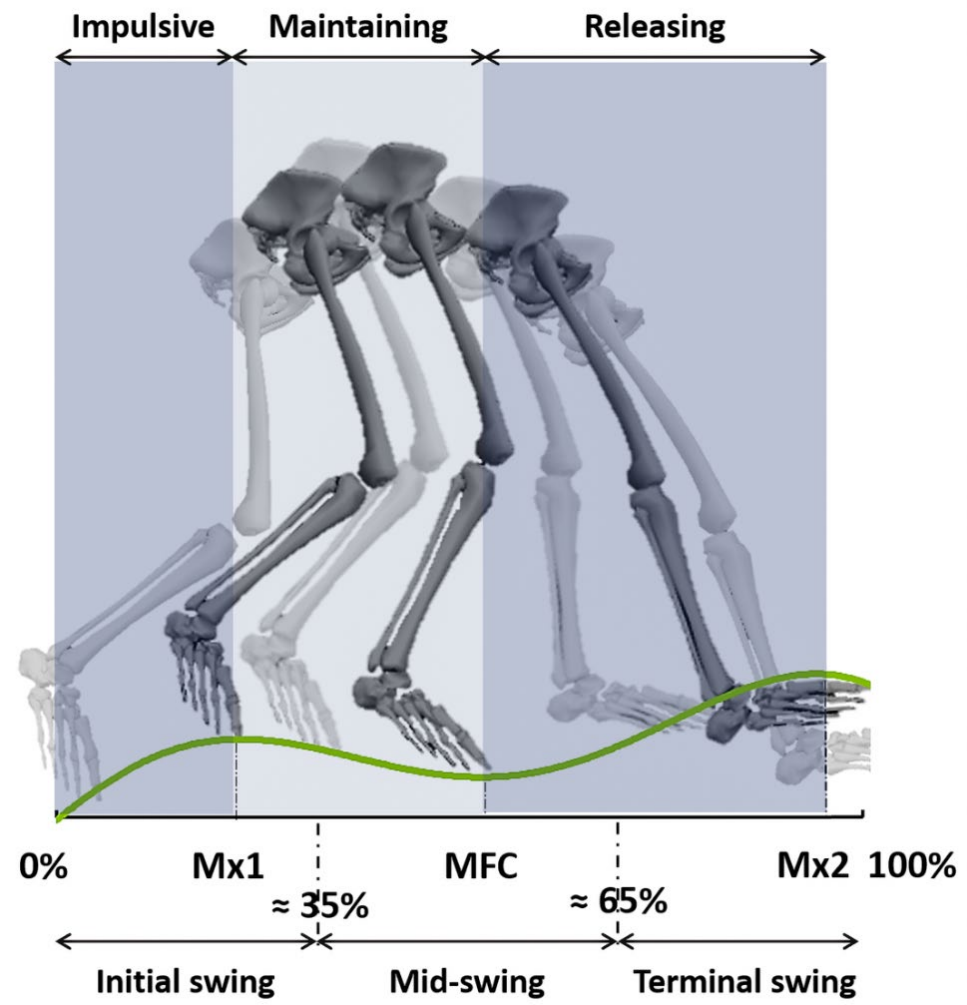
The Percentage normalized Swing sub-phases (Initial Swing ≈ 0–35%, Mid-Swing ≈ 35–65%, Terminal Swing ≈ 65–100%), versus event time-normalized sub-phases: Impulsive (Toe-off to Mx1), Maintaining (Mx1 to MFC), and Releasing (MFC to Mx2)

Gait impairments that increase the risk of tripping-related falls are one of the most serious consequences of ageing, stroke and many neurological and muscular conditions such as spinal cord injury, multiple sclerosis, muscular dystrophy or cerebral palsy [1–3]. In normal gait, the swing phase is shaped by two events, ‘Mx1’ and ‘Mx2’, representing two vertical foot displacements maxima that frame a critical moment of Minimum Toe Clearance (MTC) or Minimum Foot Clearance (MFC) (Fig. 1). MTC refers to the toe’s clearance above ground, while MFC measures the lowest part of the forefoot or shoe’s clearance from the ground. Avoiding contact with walking surface irregularities requires precisely modulated vertical displacement of the foot, especially at the swing phase Minimum Foot Clearance (MFC) event [4–6].

Ankle dorsiflexion is crucial for elevating the foot during swing by enabling substantial adjustments to ground clearance with relatively minor changes in ankle angles and minimal disruption to overall gait control [6–8]. The development of assistive technology for ankle joint dorsiflexion could play an important role in maintaining safe ground clearance and preventing tripping-related falls. Rapid progress has been observed in the development of ankle orthoses, employing advanced actuators to apply moments that can effectively assist impaired ankle dorsiflexion [9–11]. An essential requirement of these devices is to deliver sufficient mechanical power to ensure the necessary magnitude of ankle assistive moments. Understanding the kinetics demands of ankle joint dorsiflexion is, therefore, particularly useful for devices employing energy harvesting or utilizing lighter but also less power-demanding actuators. This understanding is the foundation for designing assistive technologies that harmonize the required ankle moments with required energy inputs.

Ankle dorsiflexion moments have been determined experimentally but more commonly in static conditions, rather than when walking. Takaiwa & Noritsugu (2008) [12] determined that 2 N.m ankle moment was required to achieve 20 degrees of ankle dorsiflexion, i.e. from − 15 degrees plantar flexion to + 5 degrees dorsiflexion. A University of Illinois design team adopted Perry and Burnfield’s (1993) [13] data to calibrate their powered AFO, employing a constant 3 N.m ankle torque throughout swing [11, 14–16]. Such time-dependent ankle moment measurements recorded dynamically are anticipated to be more useful in designing ankle assistive devices to more closely mimic natural gait. Kao and Ferris, (2009) [17] and Sawicki and Ferris, (2009) [18] used inverse dynamics to estimate ankle dorsiflexion moments at 1.25 m/s. They found a maximum ankle moment following toe-off of 0.016 N.m/Kg which decreased gradually until end of swing; with an ankle power range of -0.08 W/Kg to 0.05 W/Kg. Their study did not include ankle moment and power changes with increasing ankle dorsiflexion but this control feature may be useful in revealing the kinetics of high ankle dorsiflexion rotation to determine the required *adequate* mechanical energy input.

Consistent with the traditional focus on stance kinetics there are limited data to show ankle joint energy exchanges during swing, possibly because swing phase energy requirements are often considered less important components of lower limb joint kinetics [19, 20]. More recently it has, however, been argued that the energy consumed during swing is non-trivial, with research by Doke et al. (2005) [21] concluding that swing phase muscle activity consumes between one-quarter to one-third of total gait energy. Exploring joint work is important for understanding the mechanical energy demands of walking because joint mechanical energy is associated with the ability to perform work [22–24]. Ankle work can, therefore, be calculated to indicate the maximum energy demands of swing phase ankle dorsiflexion. In the study reported here we sought to determine the kinetic requirements of increasing swing phase ankle dorsiflexion by incorporating a treadmill-walking condition in which foot-ground clearance was manipulated via a continuous foot trajectory display. Subsequently, we derived the swing-phase profile of ankle joint moments and the power demands of augmenting ankle dorsiflexion.

Previous investigators have often described three swing sub-phases representing approximately 0–35%, 35–65%, and 65–100% of the swing cycle, corresponding to Initial, Mid, and Terminal swing, respectively (Fig. 1) [13, 25]. Unusual or pathological gaits may not, however, always be described adequately using these sub-phases [26] and investigation of time-dependent variables such as joint power may also require a more fine-grained analysis [27].

To explore functional variations in ankle energy demands with greater specificity, in this study we introduced three new event-dependent swing sub-phases and also calculated the time and power demands of each (Fig. 1).

In addition to determining ankle joint mechanics, foot-ankle computational modelling has been used to quantify force and power of the Tibialis Anterior (TA) as the primary dorsiflexor. A systematic review of twelve studies indicated maximum swing TA forces ranging from 1 to 4 N/kg at preferred walking speed [28] but there are important variations within sub-phases. Błażkiewicz (2013) [29] found a maximum TA force of 2 N/kg following toe-off and TA power computed by Bogey et al. (2010) [30] reached an initial negative peak of almost − 2 Watts, followed by a positive maximum of 12 Watts; those data were, however, time-normalized to the swing cycle, precluding a post-hoc work calculation. Possibly because the TA is the primary dorsiflexor, less research attention given to other ankle dorsiflexor muscles, i.e., Extensor Digitorum Longus (EDL) and Extensor Hallucis Longus (EHL). In addition to providing a more complete description of dorsiflexor kinetic contributions to ankle swing phase control, in this experiment the kinetic contributions of these three muscles were also derived.

Our objective in this study was to investigate ankle joint moments, dorsiflexor muscle forces and mechanical energy requirements of increasing swing phase ankle dorsiflexion, specifically at the high-risk Minimum Foot Clearance (MFC) event. By experimentally manipulating foot-ground clearance using a continuous feedback display, the timing and magnitude of ankle dorsiflexor moments, forces and work were modelled in response to a controlled increment in ankle dorsiflexion. It was hypothesized that relative to an unconstrained-walking control condition, greater ankle dorsiflexion would require higher ankle moments and power with increased dorsiflexor muscle forces and work.

Our findings on ankle joint and dorsiflexor muscle kinetics offer critical insights for enhancing ankle assistive technologies. Rather than increasing ankle moments, our study suggests that assistive devices should focus on providing higher mechanical energy for effective dorsiflexion. This is particularly vital for bio-inspired devices incorporating artificial muscles, where accommodating the co-activation of Tibialis Anterior and Soleus at higher dorsiflexion angles is key. These insights aim to guide the development of more efficient ankle orthoses and exoskeletons.

## Methods

### Participants

Eight healthy, physically active males (age: 35 ± 4 y; height: 175 ± 5.6 cm; mass: 78 ± 8.9 kg) were recruited. All participants undertook informed consent procedures mandated and approved by the Victoria University Human Research Ethics Committee and were screened using a health questionnaire to confirm no orthopedic, respiratory, or cardiac conditions that would preclude participation.

### Instrumentation

Three-dimensional position-time coordinates of body segments were captured using a Vicon motion capture system (Vicon, Oxford, UK), equipped with 14 Bonita cameras sampling at 100 Hz. Foot-ground reaction forces were sampled at 1000 Hz using a time-synchronized AMTI dual plate force-sensing treadmill (AMTI, MA, USA). Thirty-one retro-reflective markers, in addition to TIB and THI marker clusters, were attached to anatomical landmarks using the Vicon Plug-in-Gait marker conventions [31].

Tibialis Anterior (TA) activity was recorded using a 16-channel EMG system sampling at 1000 Hz via a Telemyo 2400T wireless transmitter (Noraxon, Scottsdale, USA). Activity of the Soleus muscle, an uniarticular ankle plantarflexor, was also recorded for model validation. Skin preparation, electrode placement and recording procedures followed the European recommendations for Surface Electromyography for the Non-Invasive Assessment of Muscle (SENIAM) [32]. EMG signals were band-pass filtered (10–500 Hz), full wave rectified, low-pass filtered (10 Hz) and normalized to maximum activation.

### Experimental procedure

**Fig. 2:**
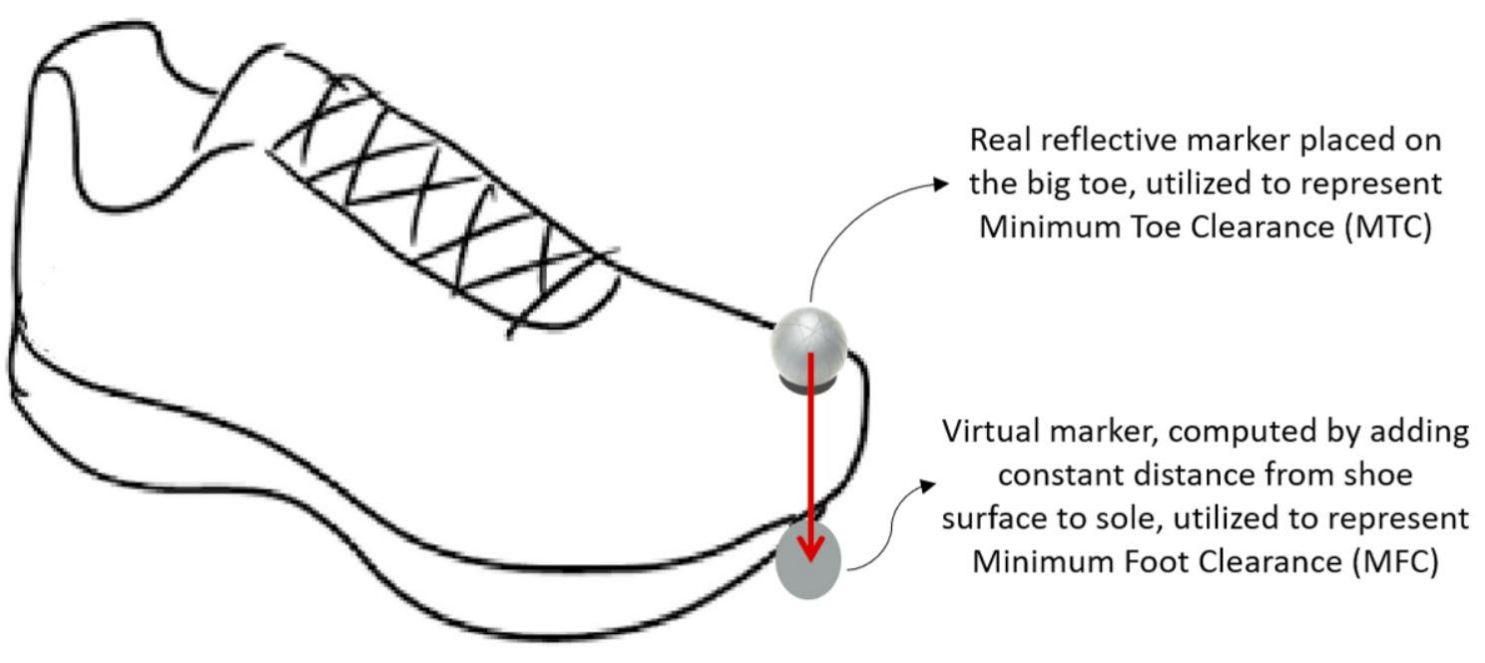
(**a**) The real marker, placed above the big toe, to control real-time monitoring of Minimum Toe Clearance (MTC) (**b**) The virtual marker, defined by adding the constant distance between the surface of the shoe and sole, to represent Minimum Foot Clearance (MFC)

A real-time feedback technique was used to control foot elevation, in which the real-time vertical displacement of dominant limb’s big toe marker was displayed on a digital monitor to indicate the target ground clearance [33, 34]. The same comfortable footwear was provided to all participants. MTC is often used to describe the trajectory of a point above the big toe while MFC commonly refers to a point beneath the shoe at the lowest part of the forefoot [35]. Consistent with Loverro et al. (2013) [35] who recommended MFC for investigating the probability of foot-ground contact a point above the big toe was used to control foot trajectory (Fig. 2). In subsequent analyses, however, a Visual 3D pipeline was developed to locate the forefoot low-point virtual marker by adding the constant distance between the surface of the shoe and sole to represent foot-ground clearance, i.e., Minimum Foot Clearance (MFC).

Each participant’s MTC was first determined using two minutes of preferred speed walking to serve as a baseline reference for the subsequent experimental MTC manipulation in which an upper boundary was defined by incrementing the participant’s baseline MTC by 4.5 cm to ensure a minimum additional 4 cm elevation, the highest MFC considered achievable using only ankle dorsiflexion [36, 37]. The choice of a 4 cm elevation for the experiment was confirmed by preliminary findings from our pilot study, demonstrating that it is the upper limit most participants could achieve through ankle dorsiflexion alone, ensuring the preservation of their natural walking pattern.

Using only an Ankle strategy and not engaging either the knee or hip (“hip hiking”), participants then walked for a further two minutes with instructions to match their dominant limb MTC with the target presented on the display monitor as a line parallel to the abscissa.

### Musculoskeletal modelling and simulation

**Fig. 3:**
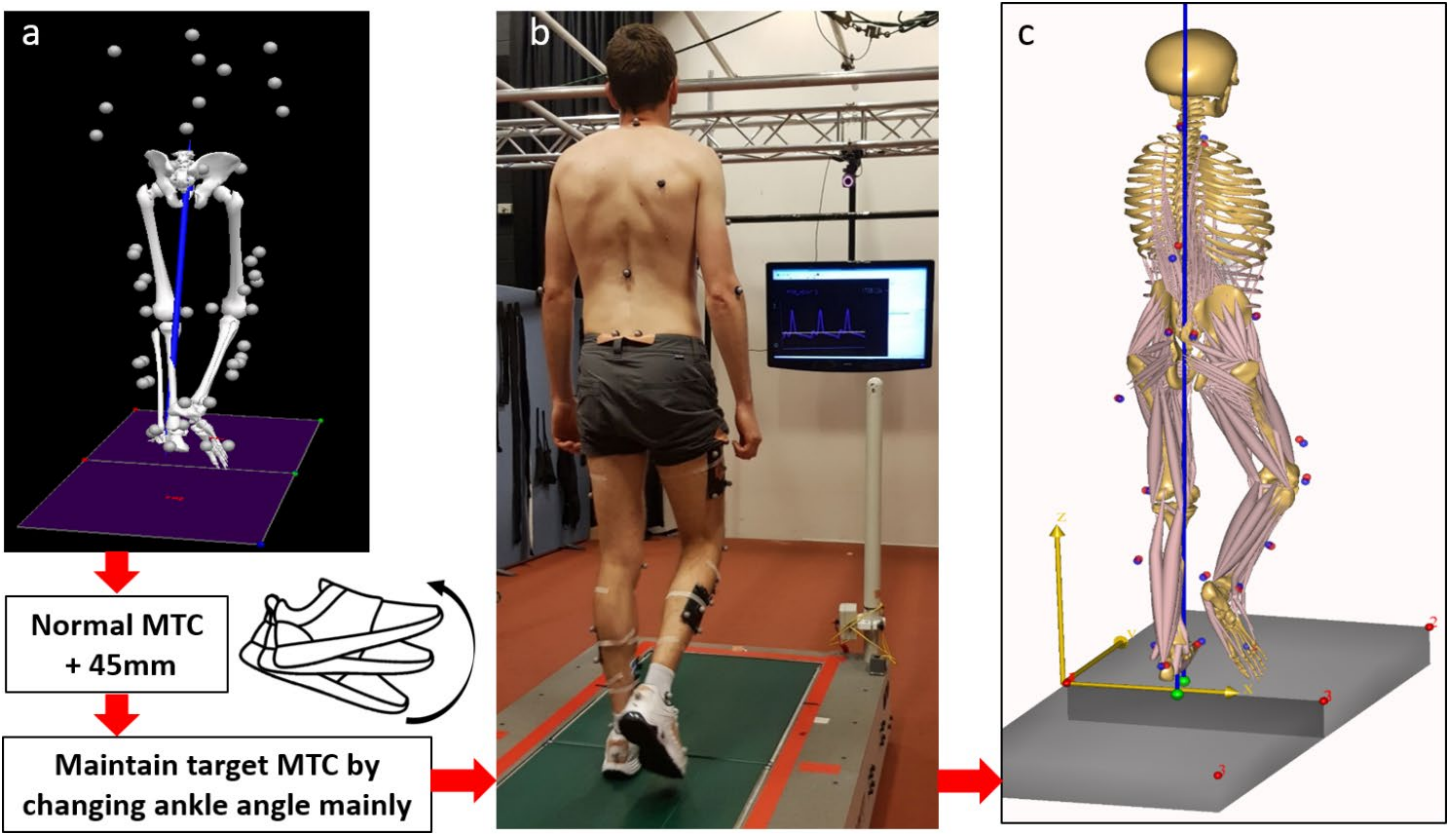
Dual belt tandem force-sensing treadmill walking task with motion captured from thirty-one Vicon reflective markers and muscles activities recorded by EMG electrodes, as described in the Methods. (**a**) Normal walking mean MTC was computed and each participant’s target MTC defined by adding 4.5 cm, using Visual 3D. (**b**) Real-time sagittal trajectory of the toe marker presented on a monitor with participants asked to match their dominant limb MTC with the displayed target using ankle dorsiflexion. (**c**) AnyBody musculoskeletal modelling with experimental markers (blue) matched to the model virtual markers (red) using an inverse kinematics simulation

Sixteen participant-specific musculoskeletal models (8 participants X 2 conditions) were developed using the AnyBody Modelling System (AnyBody Technology, Aalborg, Denmark, Version 6.0) by employing an existing generic model `MoCapModel’ (Managed Model Repository-version 1.6.3). Arms were excluded and the lower body model switched to the Twente Lower Extremity Model (TLEM) compromising foot, talus, shank, patella, thigh and hip segments [38]. Kinematic scaling was performed using the least squared minimization algorithm developed by Andersen et al., (2010) [39] by which the virtual markers assigned to the model (red points in Fig. 3c) were fit to experimental markers (blue points in Fig. 3c), to specify anthropometric parameters and the local segment coordinates for each participant. Inverse kinematics analysis was then used to compute time-histories of joint angles, using the over-determinate kinematic solver developed by Andersen et al., (2009) [40].

**Fig. 4:**
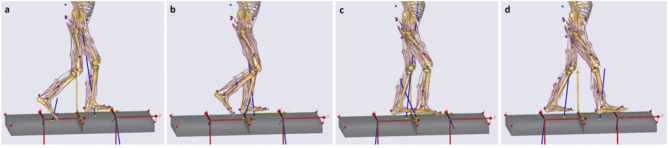
One swing cycle with successive foot-ground contacts on a dual belt tandem force-sensing treadmill, showed from toe-off of right foot (**a**) to heel-contact (**d**). Two grey shaded tandem plates are shown with their local origins (red) in which blue lines illustrate the ground reaction force vectors of each plate. Four challenging events of GRF assignment to each limb with the correct timing of foot contact with each plate assignment are shown (**a**, **b**, **c** and **d**). Figures (**a**) and (**d**) demonstrate events at which each foot contacts the anterior or posterior plate separately. The developed model algorithm detected which foot (right or left) touches anterior or posterior plate continuously. Figures (**b**) and (**c**) show events when a foot (right or left) travelling from the anterior to the posterior plate during mid-stance and the algorithm could assigned both force plates to one foot only

In overground walking, force-event synchronization is less problematic because each foot lands on each plate separately but there are some challenges regarding model adaptation for walking on a dual belt tandem (end-to-end) force-sensing treadmill as each foot contacts the anterior plate first and then moves passively onto the posterior plate. (Fig. 4). It was, therefore, necessary to differentiate limb-plate contacts during double support (Fig. 4a, d) (i.e., left or right foot) and isolate these data from simultaneous single support foot contacts (Fig. 4b, c). A computational method was devised to reliably assign tandem treadmill GRF components to each limb using an algorithm that identified the correct foot-plate contact from the captured forefoot horizontal velocity and vertical height data. During double support (Fig. 4a, d), the lead foot (left or right) was identified when the foot’s horizontal velocity changed from positive (anterior) to negative (posterior) at heel-strike. Similarly, trail foot toe-off was determined when horizontal velocity shifted from negative to positive. The foot’s vertical displacement relative to the system origin was also included to confirm that the algorithm applied when the feet were in contact with the treadmill belt. When both feet contacted the same plate simultaneously, in double support, GRF data could not be assigned and these data were excluded.

During single support at mid-stance (Fig. 4b, c) the stance foot travels from the anterior plate to posterior, the detection algorithm correctly assigned the GRF data from both plates using the constraint that the stance foot’s horizontal velocity is equal to belt speed and vertical displacement above the ground reference is minimal. Using these procedures, one complete step cycle of clearly identified GRF data were used for the normal walking simulation. In the Ankle strategy condition, in addition to the above criteria, one step that achieved the target MFC (+ 4 cm) was selected for analysis.

To run the inverse dynamics simulations the implemented Hill-type three-element muscle model was incorporated into the scaled model [41] with the muscle redundancy problem solved using the min/max optimization criterion by which the maximum force of each muscle was minimized to ensure least muscle fatigue [42].

### Data analysis

#### Kinetic variables and swing sub-phases

As shown in Fig. 1 three time-dependent sub-phases were introduced based on work by Nagano et al. (2011) [5]; Impulsive (Toe-off to Mx1), Maintaining (Mx1 to MFC) and Releasing (MFC to Mx2). The Impulsive sub-phase is associated with the rapid muscle reactions at stance termination required in the transition to swing, during the Maintaining sub-phase muscle activation is necessary to maintain the foot-ground clearance provided by the previous Impulsive sub-phase. Finally, in the Releasing sub-phase following MFC, muscle activation must be sufficient to provide controlled foot-ground contact by releasing the potential energy gained by elevating the foot.

To characterize the trajectory control characteristics of each sub-phase the experimental kinematic data were combined with the simulated ankle moments to calculate ankle power (the product of joint moment and angular velocity) and ankle work (the power/time integral within each sub-phase). Tibialis Anterior (TA) muscle work was similarly calculated using the power/time-histories exported from AnyBody.

#### Statistical analysis

Statistic Parametric Mapping (SPM) was used to analyze Ankle strategy effects on temporally normalized variables throughout swing. SPM was executed in Matlab (R2018b, Mathworks Inc., Natick, MA, USA) using open-source spm1d code (v.M0.1, www.spm1d.org [43]). To identify significant differences in gait variables between the two walking conditions curve analysis was conducted, suprathreshold areas identified and SPM two-tailed paired t-tests used to compare the normal walking and Ankle strategy group mean (*n* = 8) foot trajectory, ankle moment, ankle angle and dorsiflexor muscle forces (i.e., TA, EDL and EHL). Two-tailed paired t-tests (SPSS, Version 22, Chicago, IL, USA) were employed to test for differences in work done by the ankle joint and TA muscle within our three swing sub-phases and the complete swing phase.

## Results

**Fig. 5:**
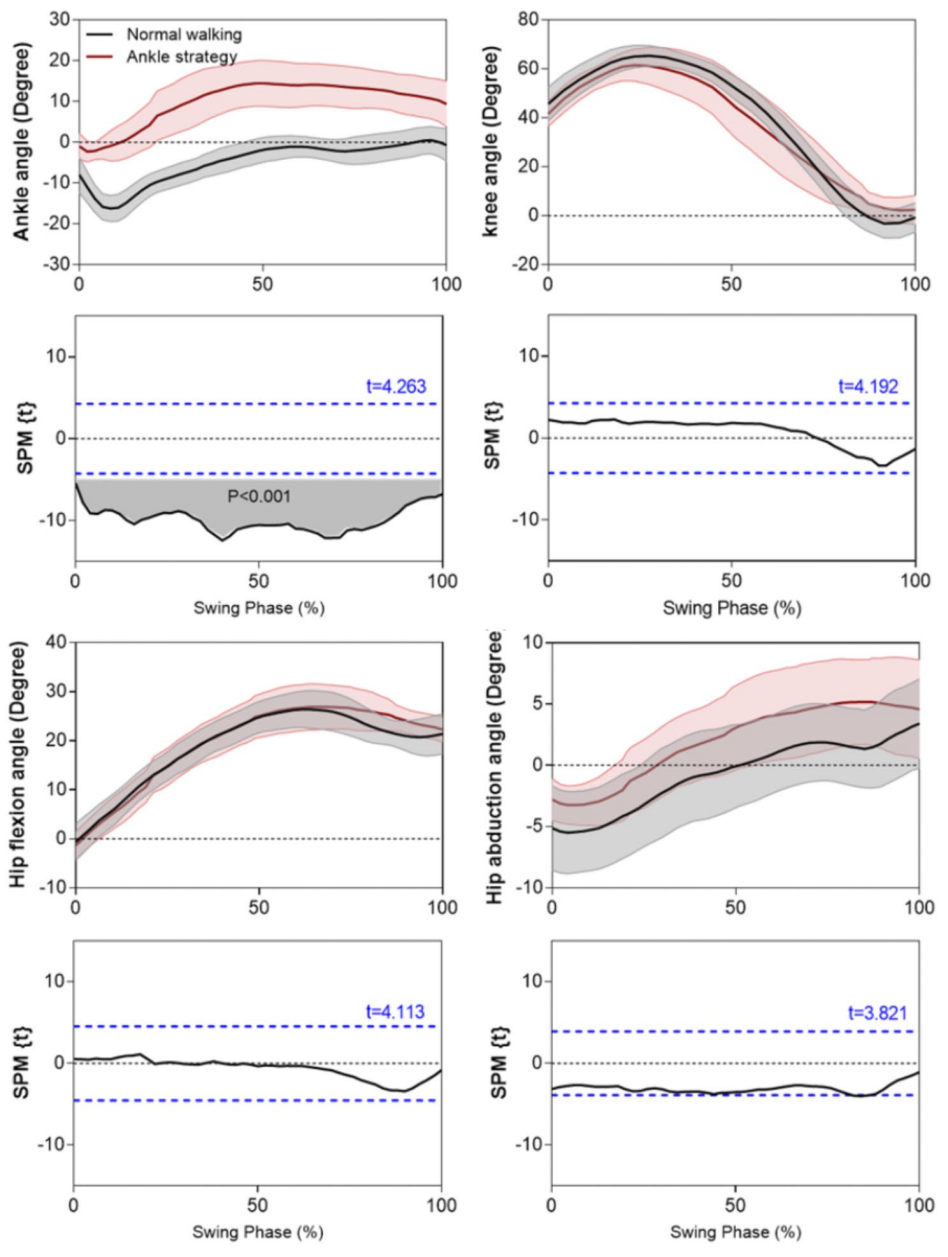
The mean (with shaded areas +/- 1 SD) lower limb joint angles for normal and Ankle strategy conditions with positive angles assigned to dorsiflexion, flexion and abduction. Shaded area SPM paired t-test analysis and dashed-line critical thresholds t values

The ankle angles in Fig. 5 show that MFC increments were associated with greater dorsiflexion throughout swing which was confirmed by the SPM paired t-tests, showing that the obtained t-value for the supra-threshold cluster within 0–100% of swing duration, exceeded the critical threshold = 4.263 (*p* < 0.001). Knee flexion, hip flexion and hip abduction were not, however, significantly affected by the Ankle strategy, confirming that the experimental procedure increased foot elevation using ankle dorsiflexion only.

**Fig. 6:**
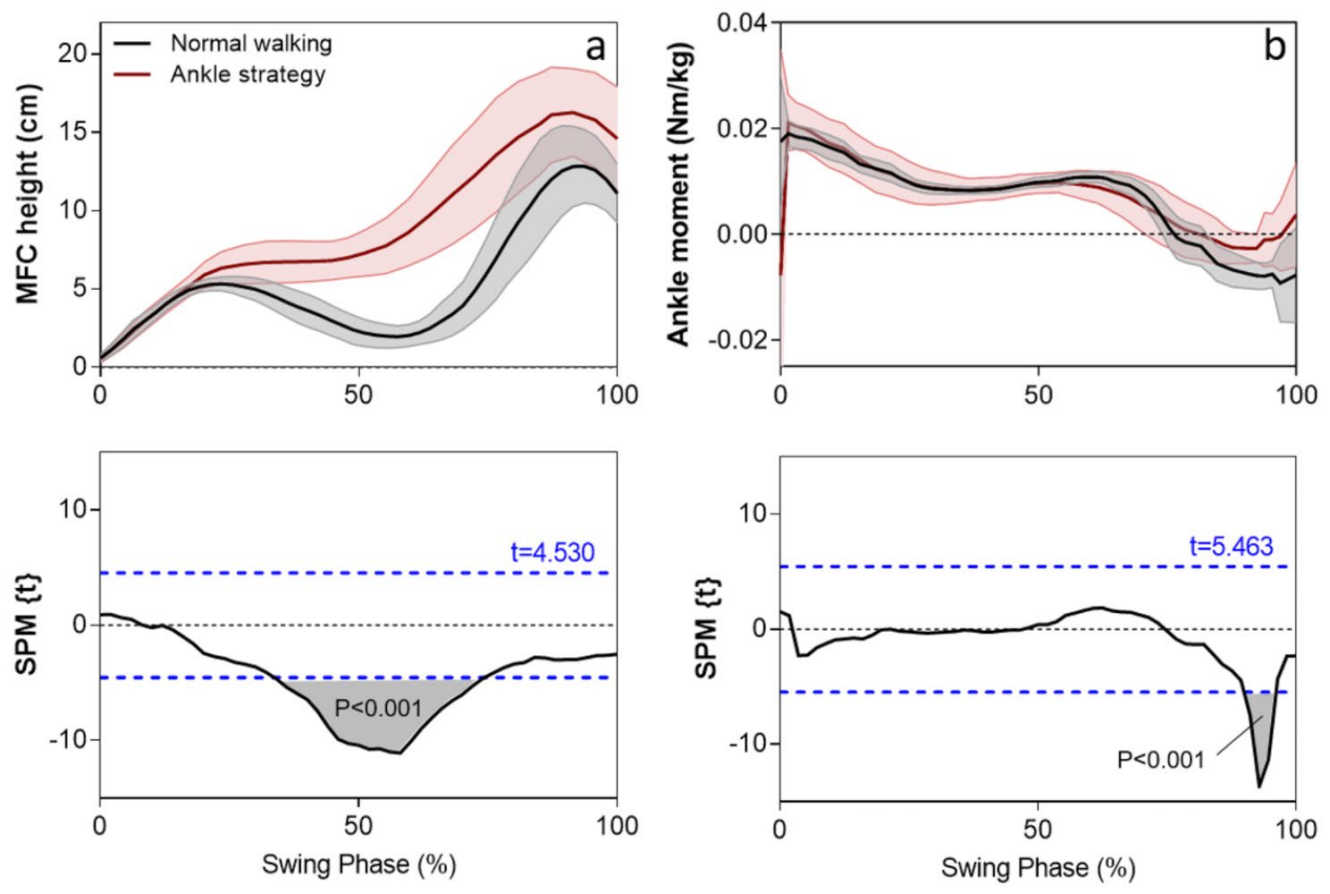
Top panels: mean +/- 1 SD swing phase time-normalised foot vertical displacement (left) and ankle moment (right) for normal walking and the Ankle strategy. Lower panels: paired t-test SPM analysis with grey shading indicating time intervals of significant (*p* < 0.05) difference between normal walking and Ankle strategy conditions. The critical threshold t values are shown with dashed lines

Foot vertical displacement during swing, shown in Fig. 6a, indicated that the target MFC increment of approximately 4 cm was achieved using the biofeedback-guided ankle control procedure (Fig. 6a). The ankle dorsiflexion strategy also affected toe-height qualitatively, elevating the toe either side of mid swing and attenuating the characteristically low ground clearance (34–74%, *p* < 0.001).

**Fig. 7:**
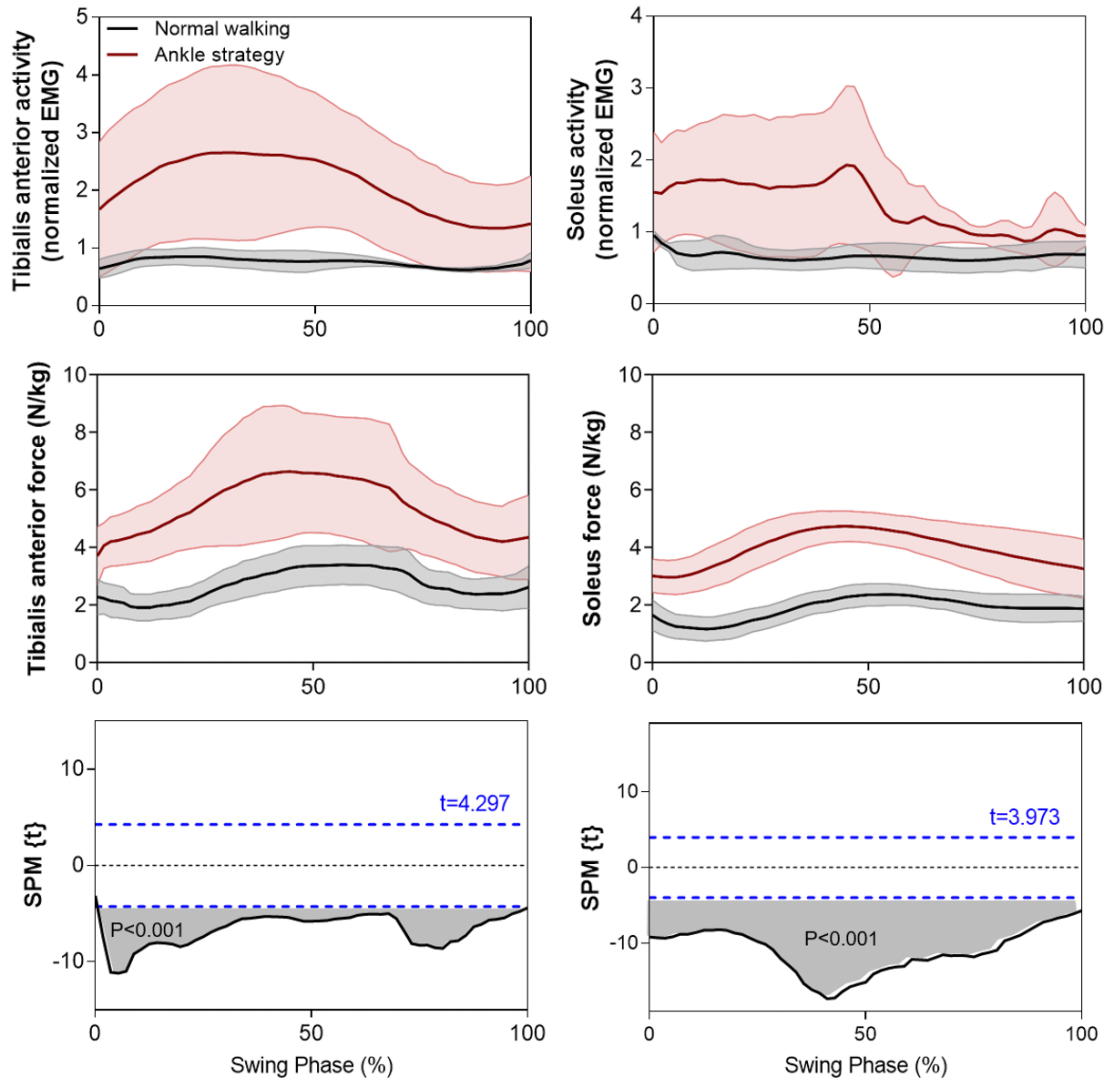
The mean + SD time-histories of TA and Soleus swing force during normal walking and the Ankle strategy compared with EMG signals normalized to maximum activation. The paired samples t-test statistic SPM {t} results indicate timing periods showing significant (*p* < 0.05) differences of TA and Soleus muscle forces (grey shaded areas). The critical thresholds (t values) are shown with a blue dashed line

**Fig. 8:**
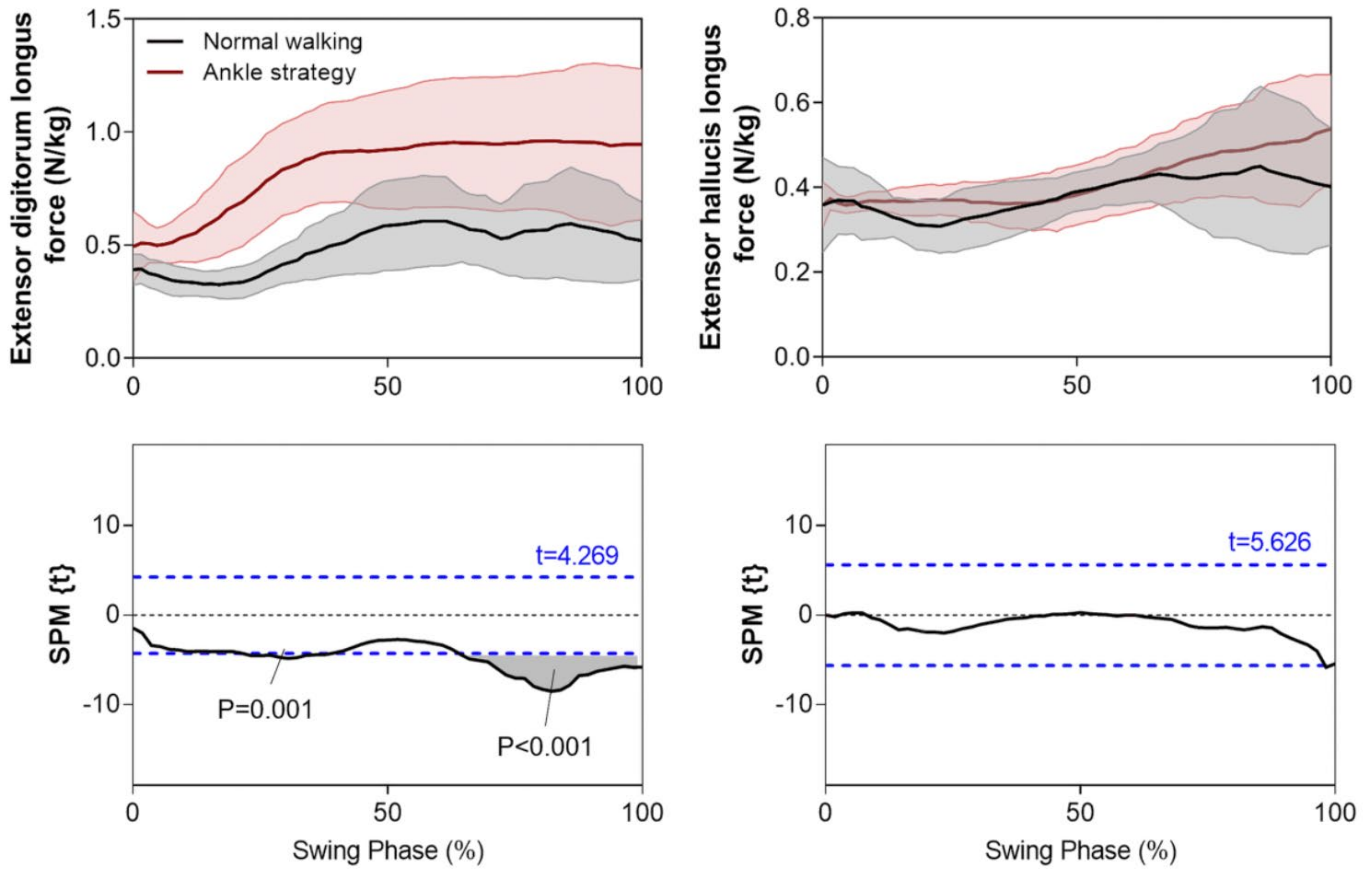
The mean + SD time-histories of average EDL and EHL swing phase muscle forces during normal walking and the Ankle strategy. The paired samples t-test statistic SPM {t} results indicate timing periods showing significant (*p* < 0.05) differences (grey shaded areas). The critical thresholds (t values) are shown with a blue dashed line

Simulation results (Fig. 6b) indicated that ankle moments using the Ankle strategy were similar to control walking, except for a short interval within terminal swing (88–95%, *p* < 0.001). Data presented in Fig. 7, however, supported the hypothesis that Tibialis Anterior (TA) force would increase using the Ankle strategy throughout swing (0–100%, *p* < 0.001) which was validated by the measured TA EMG signals. The simulated Soleus force and measured EMG signals also showed a similarly increasing pattern. The TA-Soleus co-contraction seen here is important in explaining why ankle moment did not increase in the Ankle strategy condition, as discussed further below. Extensor Digitorum Longus (EDL) force increased only within the second (21–38%, *p* = 0.001) and final quartile (67–100%, *p* < 0.001) of swing, while Extensor Hallucis Longus (EHL) force was unchanged throughout swing, re-emphasizing the TA muscle’s role in ankle dorsiflexion during swing (Fig. 8).

**Fig. 9:**
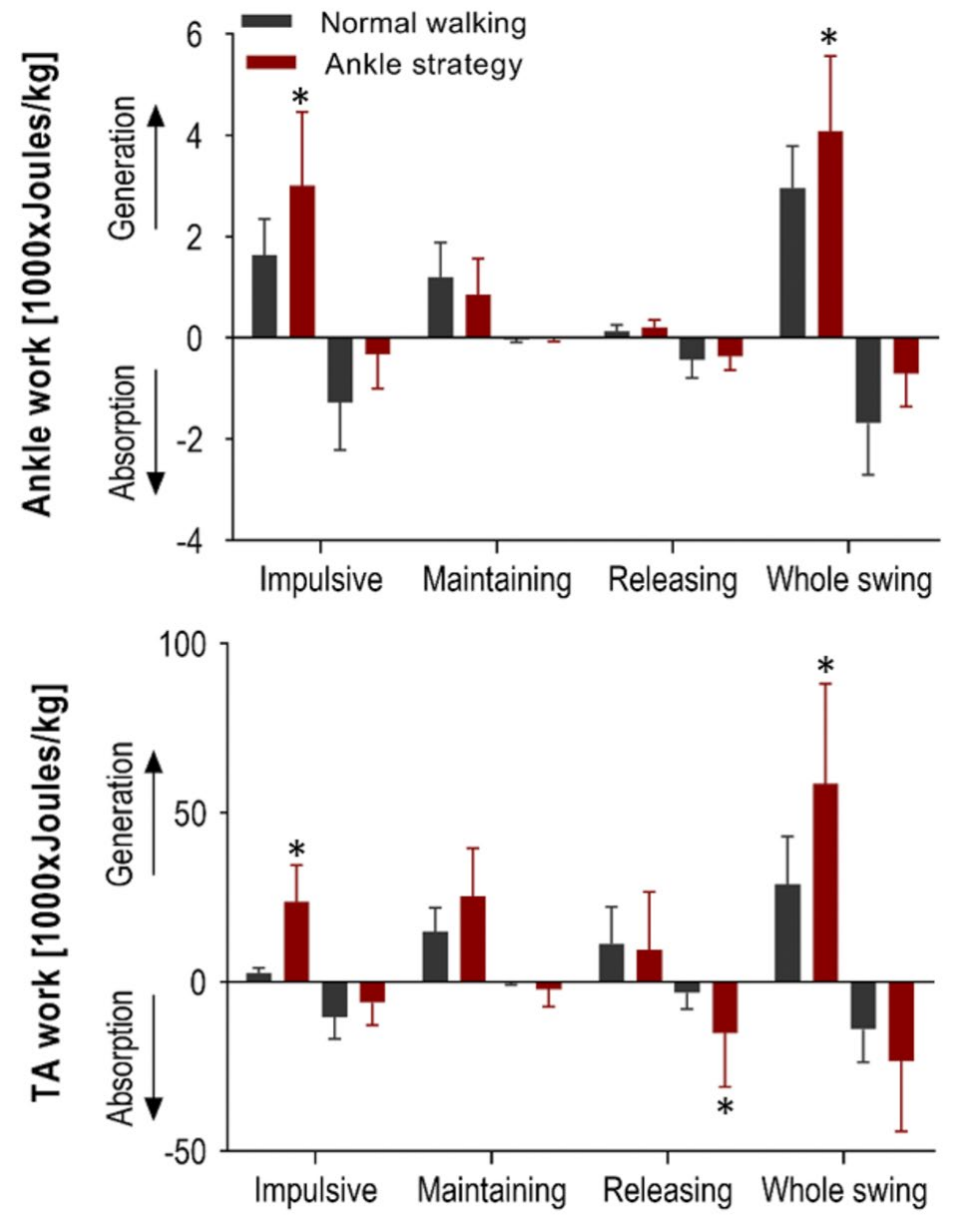
Mean (+ SD) ankle and TA work generated and absorbed within the Impulsive, Maintaining and Releasing sub-phases and whole swing with significant differences between normal walking and Ankle strategy conditions starred (*)

As indicated in Fig. 9, ankle work was greater in the Ankle strategy than for unconstrained walking during the Impulsive sub-phase (t = 2.828, *p* = 0.0225) and whole swing (t = 2.975, *p* = 0.0207). TA concentric work also increased during the Impulsive sub-phase (t = 5.595, *p* = 0.0008) and whole swing (t = 2.364, *p* = 0.0109) but only the Releasing sub-phase showed greater work absorption (t = 2.364, *p* = 0.0249) than for normal walking. In summary, greater energy is required immediately after toe-off to initiate swing with augmented ankle dorsiflexion.

## Discussion

The ankle joint is fundamental to safe and efficient locomotion by assisting foot-ground clearance, specifically at the high risk MFC event [7, 8]. This study determined the ankle kinetics required to significantly increase swing phase ground clearance (by approximately 4 cm) using real-time biofeedback [33], while knee flexion, hip flexion and hip abduction angles were unchanged from the control condition.

Our musculoskeletal model for tandem force-plate treadmill walking was substantiated by demonstrating that the simulated time-histories of Soleus and TA muscle forces during normal walking were similar to those reported in a comprehensive review of musculoskeletal modeling [28]. In addition, the measured TA and Soleus EMG activation patterns during normal walking were also consistent with published reports [30, 44–48]. Results of joint moments and powers for normal walking were also supported by published data [49–52]. Contrary to our hypothesis, no difference in ankle moments was observed between the Ankle strategy and control walking. Muscle modeling showed, however, that the Ankle strategy increased TA activity throughout swing, with a proportionate increase in TA force. This outcome was further supported by EMG data showing that TA activity exhibited a proportional change, confirming that increased TA force is necessary to facilitate foot elevation via extended ankle joint dorsiflexion. EDL and EHL muscle forces, however, did not demonstrate significant increases in activation, except for the EDL within a short period of terminal swing.

To understand why TA force increased but ankle moment did not change, the role of plantarflexor muscle action during swing (i.e. Soleus) was investigated. The Soleus swing contribution showed increased force and muscle activation, suggesting that increased TA muscle forces due to dorsiflexion may be associated with Soleus-supplemented plantarflexion forces, as seen in the EMG data. This coactivation may, therefore, have maintained ankle moments unchanged. Similar TA-Soleus co-activation has been shown in obstacle crossing; Ma et al. (2017) [53], for example, observed increased ankle antagonist coactivation with increasing obstacle height. Ankle muscle coactivation during swing can also be influenced by medical conditions and Lee (2020) [54] found reduced ankle muscle coactivation for patients with incomplete spinal cord injury, while Ma et al. (2017) [53] showed greater coactivation for stroke survivors compared to healthy controls.

The above findings reinforce the importance of considering ankle dorsiflexor/plantarflexor coactivation prior to designing interventions to assist weakened dorsiflexors. It is reasonable to conclude that interventions to assist ankle dorsiflexion *may* require a plantarflexor muscle contribution but further investigation is required to determine whether this co-activation is seen when an active external assistive device is used to provide a TA-assisting force.

Results of total work computations supported the hypothesis that the Ankle strategy would require more energy than control walking. Ankle work computations within the three swing sub-phases showed that the Ankle strategy involved greater total work, with additional energy required immediately following toe-off, in the Impulsive sub-phase. It can, therefore, be concluded that an impulsive force is required in a very short time to effect the transition from plantarflexion after toe-off, to dorsiflexion [55, 56]. TA-Soleus coactivation may, furthermore, also explain the increased TA work required for ankle dorsiflexion throughout swing but particularly within the Impulsive sub-phase.

The time-synchronized kinetic data and associated dorsiflexor muscle activity presented here could be highly informative in optimizing the timing and magnitude of forces provided by an ankle assistive device. In recent developments in assistive devices using artificial muscles, TA force synchronization with ankle moments is necessary to ensure reliable simulation of living-muscle activity [18, 29, 57]. Lee and Hogan (2014) [58] demonstrated that TA activation mirrors the ankle moment, with a peak after toe-off and activation decreasing up to the end of swing. Kao and Ferris (2009) [17] also used the TA activation envelope in designing their artificial muscle-powered device but did not calculate TA kinetics to illustrate the associated force and work. In the present study, ankle joint and TA muscle kinetics, including force and work, were computed to supplement our understanding of the time-dependent kinetic demands of ankle dorsiflexion during swing. Unchanged EDL and EHL ankle dorsiflexor muscle forces confirmed that the TA is essentially responsible for swing phase ankle dorsiflexion.

The energy required in elevating the foot can be determined by evaluating the work related to joint power [59]. Our estimate of the energy demands of augmenting ankle dorsiflexion suggest that an ankle assistive device may not need to generate significantly greater ankle moments than during normal unconstrained walking. Nevertheless, a noteworthy design consideration involves an energy infusion immediately following toe-off. These finding suggest that either a passive ankle-assistive device harnessing recovered energy or a low-power mechanical ankle-actuator [60–62], would be required to generate up to 0.003 Joules/kg immediately following toe-off to produce the requisite 0.02 N.m/kg ankle moment. Ankle-related assistive devices [63] with TA artificial muscles may also require an application of approximately 0.025 Joules/kg after toe-off to provide a 4 N/kg force, necessary, in part, to overcome plantar-flexor muscle co-contraction. Ankle dorsiflexion energy will, however, be influenced by parameters such as specific muscle weakness or spasm, which must be investigated by modelling the swing phase kinetics of gait-impaired populations [64, 65].

Due to the challenges associated with direct measurements, in this project computerized simulation was used to investigate the ankle joint’s swing phase kinetics. There are, however, limitations to this approach due to soft-tissue artefacts [66, 67], muscle modelling assumptions [68] and optimization methods [69]. Our findings in this study, however, showed a good match between TA and Soleus EMG signals and muscle forces, achieved using the kinematic optimization [66], the min/max muscle activity optimization criterion [42] and a Hill-type three-element muscle model [41]. There is no direct relationship between EMG signals and muscle forces [28, 70] and in this experiment we used EMG data only for model validation. Additionally, the exclusion of female participants in this study, necessitated by recruitment challenges during the COVID-19 pandemic and the specific biomechanical focus of our research, represents a limitation we acknowledge. Future studies will aim to include a more diverse participant pool, additional empirical measurements, and further analyses to enhance our understanding of ankle dynamics. These efforts will also help confirm the role of ankle flexor muscles and explore the contributions of knee and hip mechanics in controlling swing phase trajectory.

## Conclusion

This study described the kinetics of swing phase ankle dorsiflexion, highlighting the interplay of ankle moments, muscle forces, and energy demands, with a particular emphasis on understanding the required minimum kinetic demands. It was hypothesized that significantly greater ankle moment, mechanical energy and dorsiflexor muscle forces would be required to increase foot-ground clearance using heightened ankle dorsiflexion. Our findings did not confirm increased ankle moments but there was support for increased TA force and energy demand.

It is concluded that external assistive devices designed to increase ankle dorsiflexion will not be required to provide a significantly greater moment. Instead, these devices should be engineered to deliver adequate mechanical power to facilitate rapid energy bursts, particularly before reaching critical event of Minimum Foot Clearance (MFC). Additionally, a supplementary TA muscle force, due to Tibialis Anterior and Soleus co-activation at higher ankle dorsiflexion angles, should also be considered a requirement for bio-inspired technologies like biomimetic artificial TA muscles or humanoid robots.

Our detailed analysis of energy requirements across swing sub-phases offers guidance for developments in assistive power sources for passive devices and low-power actuator technologies, ensuring they enhance mobility effectively while accommodating the biomechanical realities of human movement.

## Data Availability

The datasets used and analysed during the current study are available from the corresponding author on reasonable request.
